# A85 LIKELIHOOD OF ENDOSCOPY CANCELLATION BASED ON VANCOMYCIN-RESISTANT ENTEROCOCCI (VRE) COLONIZATION STATUS AND INFECTION CONTROL PRACTICES

**DOI:** 10.1093/jcag/gwab049.084

**Published:** 2022-02-21

**Authors:** J G Lee, G Ou

**Affiliations:** 1 ^1.^ University of British Columbia, Vancouver, BC, Canada; 2 ^2.^ St. Paul, Vancouver, BC, Canada

## Abstract

**Background:**

“Terminal cleaning” is a practice of rigorous cleaning of endoscopy suite following endoscopies for patients colonized with vancomycin-resistant enterocci (VRE) with the intention of reducing VRE transmission. Such practice entails double-wiping all surfaces including the floor with disinfectants before a non-VRE patient can use the endoscopy room. While intuitive, such time-consuming practice is not supported by evidence and may have unintended negative impact on patient access to timely endoscopic evaluation.

**Aims:**

To determine whether terminal cleaning of endoscopy suite for VRE-colonized patients has any negative impact on inpatient access to timely endoscopic evaluation.

**Methods:**

As part of a quality improvement study, inpatient endoscopy data was gathered over a 3-month period between February 2021 and April 2021 at a tertiary centre. EUS, ERCP, and travel cases outside of the endoscopy suite were excluded. The cancellation rates were compared between VRE-colonized patients and non-VRE patients using the Fisher’s exact test. P value of <0.05 was considered statistically significant.

**Results:**

A total of 262 inpatient endoscopic procedures were scheduled and included in the study. Sixty-six (25.2%) of inpatient procedures were cancelled during this period (Table 1). A total of 24 procedures were scheduled for VRE patients, 9 of which were cancelled because of insufficient operating time and two due to concurrent carbapenamase-producing organism carriage and poor bowel preparation. In the non-VRE group, 55 (23.3%) procedures were cancelled for various reasons (Table 1). In subgroup analysis where cancellations related to COVID-19 (n=14) were omitted, VRE patients had a significantly higher rate of procedure cancellations compared to non-VRE patients (42.3% vs. 18.5%; p<0.01).

**Conclusions:**

The overall endoscopy cancellation rate for VRE-colonized patients was higher than those who were non-VRE-colonized. We propose that this is likely secondary to the delays from unnecessary terminal cleans imposed for VRE-colonized patients and await for post-intervention data.

**Funding Agencies:**

None

**Table:** Table 1: Rate of endoscopy cancellation for VRE and non-VRE colonized patients.

Total procedures listed	262
Total procedures performed	196
Total procedures cancelled	66
Procedure cancelled reason (n=66)	- VRE positive only = 9 (13.6%) - VRE and CPO positive = 1 (2%) - VRE positive and did not finish bowel preparation = 1 (2%) - COVID pending = 14 (21.2%) - Nothing specified = 40 (60.6%) - Patient declined procedure = 1 (2%)
Overall procedure cancel rate	25.2% (66/262)
Overall procedure cancel rate without COVID-19	21.0%% (52/248)
VRE procedures listed	26
VRE procedures cancelled	11
VRE procedure cancel rate	42.3% (11/26)
Non-VRE procedures listed	236
Non-VRE procedures cancelled	55
Non-VRE procedures cancel rate	23.3% (55/236)
Non-VRE procedures cancel rate without COVID-19 confounder (n=14)	18.5% (41/222)

